# Society Meetings

**Published:** 1897-05

**Authors:** 


### Society Meetings.

THE National Confederation of State Medical Examining and Licensing Boards will hold its seventh annual meeting at the Hotel Walton, Philadelphia, beginning at 10 o'clock A. M., Monday, May 31, 1897, under the presidency of Dr. William Warren-Potter, of Buffalo. The following program has been arranged by the secretary, Dr. A. Walter Suiter, of Herkimer: Papers. (1) Some practical experience with, and results of, the medical law of Pennsylvania, by William S. Foster, Pittsburg. (2) The need for exact information as to equipment, methods and requirements of our medical schools, by J. N. McCormack, Bowling Green, Kentucky. (3) Address by Professor J. W. Holland, M. D., dean Jefferson Medical College, Philadelphia. (4) Remarks on medical practice laws in the new northwest, by C. K. Cole, Helena, Montana. (5) The Alabama system, by W. H. Sanders, Montgomery, Ala.

There will be an address of welcome by Dr. A. H. Hulshizer, of the Pennsylvania state board of medical examiners, and a response thereto by vice-president Charles A. L. Reed, of Ohio; a report of the committee on minimum standards; report of the secretary and treasurer; annual address of the president; and miscellaneous and executive business will be transacted.

A cordial invitation is extended to all members and ex-members of state medical examining boards, and to physicians, sanitarians and educators, who are friendly to state control in medicine and to the advancement of the standard of medical education in the United States, to attend the meeting and participate in its proceedings.

The American Medical Association will hold its semicentennial meeting in Philadelphia on the 1st, 2d, 3d and 4th of June, 1897. This meeting bids fair to surpass, in the character of the entertainment, the scientific papers and the number in attendance, any meeting which has heretofore been held. The committee in charge has been able to obtain large and roomy places of meeting for the general and section meetings, all within a single block and within very short walking distance or immediately adjacent to the largest and most comfortable of the Philadelphia hotels.

For the week preceding and following the meeting the committee of arrangements has also arranged for clinical courses, which will be open without charge to all physicians who may visit the city at that time. These courses cover every branch in medicine and its specialties and will afford visitors the opportunity of seeing the active clinical work of all the great teachers of Philadelphia, which is now, as it has been for so many years in the past, in every respect one of the medical centers of the United States.

On the second day of the meeting, exercises to commemorate the founding of the association will be held under the supervision of the committee on anniversary exercises, Dr. John B. Roberts, chairman. The committee has invited the presidents of state medical societies and the presidents of state medical examining boards to participate in the ceremonies.

---

The Buffalo Academy of Medicine held meetings during the month of April as follows:

*Section on Surgery.*—Tuesday evening, April 6th, program: Rectal prolapse—rectopexy, by Dr. Herman Mynter; Exhibition of cases: gastrostomy, Witzel's method, by Dr. Eugene Smith.

*Section on Medicine.*—Tuesday evening, April 13th, program: Physiology and pathology of cycling—nervous system, by Dr. Herman Matzinger; Heart and lungs, by Dr. John H. Pryor; Digestive system, by Dr. Chas. G. Stockton; Pelvic Viscera, by Dr. M. D. Mann.

*Section on Pathology.*—Tuesday evening, April 20th, program: Recent progress in cellular biology, illustrated, by Professor Charles Wright Dodge, of Rochester University.

*Section on Obstetrics and Gynecology.*—Tuesday evening, April 27th, program: Abortion, etiology and pathology, by Dr.

C. S. Jewett; Diagnosis and treatment, by Dr. C. C. Frederick; A plea for uterine curettage as a routine measure after abortion before the third month, by Dr. S. Goldberg

---

The American Medical Publishers' Association will hold its fourth annual meeting at Philadelphia, May 31, 1897, under the presidency of Dr. Landon B. Edwards, editor and publisher of the Virginia semi-monthly, of Richmond. The secretary, Mr. Charles Wood Fassett, of St. Joseph, Mo., invites all who desire to read papers on that occasion to send the titles thereof to him at an early day. The membership fee, $5.00, is now payable and, likewise, should be sent to Mr. Fassett.

---

The American Public Health Association will hold its twenty-fifth annual meeting at Philadelphia, October 26–29, 1897, under the presidency of Dr. Henry B. Horlbeck, of Charleston, S. C. The secretary, Dr. Irving A. Watson, of Concord, N. H., has issued a preliminary announcement giving the topics for consideration and general information concerning the meeting. Dr. Benjamin Lee, 1532 Pine street, Philadelphia, is the chairman of the local committee of arrangements.

---

The American Institute of Homeopathy will hold its next annual meeting at Buffalo, June 23–30, 1897. The chairman of the committee of arrangements, Dr. A. R. Wright, of this city, has issued a preliminary circular giving details of the work of his committee in preparing for the meeting, and which affords much important information for the visitors. Dr. J. T. Cook is secretary of the local committee.

---

The Lake Erie Medical Society held its regular meeting at Dayton, N. Y., April 14, 1897, under the presidency of Dr. A. D. Lake, of Gowanda. Among other proceedings Dr. J. G. Thompson, of Angola, read a paper on the Management of abortion, that was discussed by Drs. Lattin, Ward and Beardsley.

---

The Missouri State Medical Association will hold it fortieth annual session at St. Louis, May 18, 19 and 20, 1897, under the presidency of Dr. J. H. Duncan, of St. Louis. A preliminary announcement has been sent out that includes a large number of interesting titles.

The American Academy of Medicine will hold its twenty-second annual meeting in parlor C of the Continental Hotel, Philadelphia, on Saturday, May 29 and Monday, May 31, 1897, under the presidency of Dr. J. C. Wilson, of Philadelphia. The secretary, Dr. Charles McIntire, of Easton, Pa., has sent out a preliminary announcement that includes a large number of valuable titles. It is anticipated that this meeting will be one of the largest and most interesting ever held by the Academy.

---

The Congress of American Physicians and Surgeons, composed of some of the national special societies, will hold its fourth triennial session at Washington, May 4–6, 1897, under the presidency of Dr. William H. Welch, of Baltimore. All physicians are invited to attend the meetings of the congress, but only those may register who are members, specially invited guests, or visitors accredited through the constituent societies.

---

The Medical Society of the State of Wisconsin will hold its fifty-first annual meeting at Racine, May 5, 6 and 7, 1897, under the presidency of Dr. D. O. Reynolds, of Lake Geneva. The secretary, Dr. Charles S. Sheldon, has sent out a classified program, embracing all the various branches of medicine. The anniversary banquet will close the proceedings on the afternoon of the third day at the Hotel Racine.

---

The American Medical Editors' Association will hold its next annual meeting at Philadelphia, June 1, 1897, under the presidency of Dr. Hobart A. Hare, of that city. The association will hold its annual dinner at the Hotel Aldine, when a large attendance is expected. Dr. H. Burt Ellis, of Los Angeles, Cal., is the secretary.

---

The Association of American Medical Colleges will hold its next annual meeting at the Hotel Walton, Philadelphia, Monday, May 31, 1897, at 10 o'clock A. M., under the presidency of Dr. J. M. Bodine, of Louisville. A new constitution will be acted upon and other important business transacted.

---

The Medical Society of the State of Pennsylvania will hold its forty-seventh annual meeting at Pittsburg, May 18–20, 1897. An interesting program has been issued and the officers are preparing for a full attendance of members.

